# Whole body magnetic resonance angiography and computed tomography angiography in the vascular mapping of head and neck: an intraindividual comparison

**DOI:** 10.1186/1746-160X-10-16

**Published:** 2014-05-12

**Authors:** Manuel Kramer, Siegfried A Schwab, Emeka Nkenke, Achim Eller, Ferdinand Kammerer, Matthias May, João F Baigger, Michael Uder, Michael Lell

**Affiliations:** 1 Institute of Radiology, Maximiliansplatz 1, 91054 Erlangen, University of Erlangen-Nuremberg, Maximiliansplatz 1, Erlangen 91054, Germany; 2 Department of Oral and Maxillofacial Surgery, University of Halle (Saale), Halle (Saale), Germany

**Keywords:** Magnetic resonance angiography, Computed tomography angiography, Carotid artery, Microvascular surgery, Head and neck cancer

## Abstract

**Introduction:**

The aim of the study was to compare the detectability of neck vessels with contrast enhanced magnetic resonance angiography (MRA) in the setting of a whole-body MRA and multislice computed tomography angiography (CTA) for preoperative vascular mapping of head and neck.

**Methods:**

In 20 patients MRA was performed prior to microvascular reconstruction of the mandible with osteomyocutaneous flaps. CTA of the neck served as the method of reference.

1.5 T contrast enhanced magnetic resonance angiograms were acquired to visualize the vascular structures of the neck in the setting of a whole-body MRA examination. 64-slice spiral computed tomography was performed with a dual-phase protocol, using the arterial phase images for 3D CTA reconstruction. Maximum intensity projection was employed to visualize MRA and CTA data. To retrieve differences in the detectability of vessel branches between MRA and CTA, a McNemar test was performed.

**Results:**

All angiograms were of diagnostic quality. There were no statistically significant differences between MRA and CTA for the detection of branches of the external carotid artery that are relevant host vessels for microsurgery (p = 0.118). CTA was superior to MRA if all the external carotid artery branches were included (p < 0.001).

**Conclusions:**

MRA is a reliable alternative to CTA in vascular mapping of the cervical vasculature for planning of microvascular reconstruction of the mandible. In the setting of whole-body MRA it could serve as a radiation free one-stop-shop tool for preoperative assessment of the arterial system, potentially covering both, the donor and host site in one single examination.

## Introduction

Microvascular grafts are often required to cover large resection defects following oral tumor surgery or osteoradionecrosis of the mandible. Their use has been well established in oral and maxillofacial surgery. One of the main factors for the success of these reconstructions is a sufficient blood supply of the graft by the host vessels. However, the perfusion of the host vessels can be impaired as a consequence of tumor infiltration, previous irradiation or surgery. Moreover, as a result of chronic nicotine abuse, many patients with cancer of the oral cavity are at a higher risk for coexisting atherosclerosis in peripheral arteries. Therefore the status of the host vessels has to be determined in order to identify vascular problems before the reconstruction procedure is performed. A detailed planning includes proof of a sufficient vessel perfusion to decide whether the host site meets the requirements for microvascular reconstruction [1].

Selective intraarterial angiography is considered the gold standard in the assessment of the carotid arteries due to excellent spatial and temporal resolution. Nevertheless the invasive nature of this procedure with a small but not negligible risk of neurological complications is a drawback of this modality. Multislice spiral computed tomography has been established as a non-invasive alternative for comprehensive imaging of the anatomy of the cervical vasculature and soft tissues [2-4]. Contrast enhanced magnetic resonance angiography (MRA) is an ideal non-invasive tool without radiation exposure for whole-body evaluation of atherosclerotic disease. Therefore whole-body MRA has been introduced recently for presurgical planning of microvascular (e.g. fibular) free-flap head and neck reconstructions [5] and could serve as a potential one-stop shop tool for preoperative assessment of the arterial system, covering both the donor and host site.

Therefore it was the aim of our study to compare the detectability of neck vessels with contrast enhanced magnetic MRA in the setting of a whole-body MRA and multislice computed tomography angiography (CTA) for vascular mapping of head and neck.

## Materials and methods

### Patients

Twenty consecutive patients, mean age 64years (range: 42–78 years), with a history of oral squamous cell carcinoma, scheduled for resection and microvascular reconstruction were included in this prospective study. Twelve patients had no previous treatment; eight patients had either tumor recurrence or osteoradionecrosis after previous radiotherapy (50–70 Gy).

All studies were conducted in accordance with the guidelines of the Declaration of Helsinki and in coordination with institutional board guidelines of the University of Erlangen-Nuremberg. Written informed consent was obtained from each patient. A dual-phase CT examination (arterial and late venous phase) is the routine procedure in the work-up of these patients at our institution. In addition to CT, all patients underwent whole body MRA prior to surgery. Patients were excluded from the study if they had contraindications for MRA or CTA (e.g. pacemaker, claustrophobia, severe renal insufficiency or history of adverse reactions to contrast material). Both examinations were performed within less than 72 hours.

### Examination protocols

#### Multislice computed tomography angiography

CT was performed with a 64-slice spiral CT scanner (Somatom Sensation 64, Siemens Healthcare, Forchheim, Germany) as described before [4]. Arterial phase as well as late phase images (t_delay_ = 80s) were acquired in order to improve both the visualisation of tumor vascularization and tumor extent. 100 ml of a nonionic contrast agent (Imeron 350, Bracco, Milan, Italy) followed by a saline flush of 50 ml were injected via an 18-gauge antecubital venous catheter with a power injector at a rate of 5 ml/s. To choose the individual start delay (t_delay_) for the arterial phase imaging, a test bolus technique (10 ml CM, 5 ml/s, saline flush of 30 ml) was used. The scan volume ranged from the skull base to C6 level, resulting in a typical scan length of about 15 cm. The scanning settings for CTA were 120 kV, 140 eff. mAs, 64 × 0.6-mm slice detector configuration; table speed was 19.2 mm/s (pitch 1.0) and gantry rotation time was 0.33 s. All examinations were performed under breath hold and the patients were instructed to avoid swallowing during the examination. Thin slices of 0.75-mm were reconstructed with an increment of 0.5 mm using a soft tissue convolution algorithm (field of view (FOV) 180 mm; matrix of 512 × 512; voxel size 0.35 × 0.35 × 0.5 mm).

Adverse reactions or complications during and after the procedure were recorded.

#### Contrast enhanced magnetic resonance angiography

MRA was performed as a whole-body examination on a 1.5-T MR scanner (Magnetom Avanto, Siemens Healthcare, Erlangen, Germany) [5]. Surface coils consisting of up to 76 coil elements that can be assigned to 32 independent receiver channels which covered the entire body. No repositioning was required during the examination. Four overlapping fields of view (FOVs) of 500 mm in read direction were used. The overlap between adjacent FOVs was 40 mm resulting in a total coverage of 1800 mm in the feet-head direction. Anatomical coverage was divided into four stations: Station I (aortic arch and the head and neck vessels), station II (thoracic, abdominal, and pelvic vessels), station III (upper leg), and station IV (lower leg). Image acquisitions of stations I and II were performed under inspiratory breath hold. Prior to the examination, an 18-gauge intravenous catheter was placed into an antecubital vein of the right arm for injection of contrast medium. After acquiring a vessel scout and determination of the circulation time with a test bolus (1 ml CM, 1 ml/s, saline flush of 20 ml) MRA was performed after injection of a total of 0.1 ml/kg body weight (bw) gadobutrol (Gadovist, Bayer HealthCare, Leverkusen, Germany) at a rate of 1 ml/s followed by a saline bolus of 20 ml. After acquiring the non-enhanced baseline images of station I-IV MRA at station I and IV was performed using 50% of the contrast material followed by 20 ml saline. Thereafter stations II and III were acquired using the remaining 50% contrast material. For sequence parameters see Table 1.

**Table 1:** Sequence parameters for contrast enhanced whole-body magnetic resonance angiography

**Parameter**	**Station I**	**Station II**	**Station II**	**Station IV**
TR (ms)	2.6	2.9	3.6	4.0
TE (ms)	1.0	1.0	1.3	1.3
Flip angle (°)	25	21	25	30
Band width (Hz)	620	440	340	290
Slice thickness (mm)	1.2	1.6	1.4	1.2
Slices per slab	88	72	72	96
FOV (mm^2^)	500 x 328	500 x 375	500 x 328	500 x 328
Parallel acquisition	PAT 2 (GRAPPA)	PAT 2 (GRAPPA)	PAT 2 (GRAPPA)	PAT 2 (GRAPPA)
k-space sampling	Linear	Linear	Centric	Centric
Voxel size (mm^3^)	1.1 x 1.5 x 1.2	1.0 x 1.6 1.6	1.0 x 1.4 x 1.4	1.0 x 1.2 x 1.2
Scan time (s)	19	13	15	26

To improve vessel to background contrast, baseline images were subtracted from the contrast-enhanced images. As with CTA any adverse reactions or complications were noted.

### Image evaluation

The assessment of the branches of the external carotid artery in the two imaging modalities was performed in a randomized fashion on a workstation by two radiologists with broad experience in both head and neck imaging and MRA in consensus. Evaluation sessions for MRA and CTA were performed at least 4 weeks apart to avoid possible recall bias. Only station I (for comparison with CTA of the neck) and station IV (for evaluation of the donor site in the lower leg) from the whole-body MRA was evaluated in this study. The visualization of the carotid bifurcation and the major branches of the external carotid artery (external carotid artery mainstem, superior thyroid artery, lingual artery, facial artery, ascending pharyngeal artery, occipital artery, posterior auricular artery, superficial temporal artery and maxillary artery) were recorded for MRA and CTA separately. At the potential donor sites in the lower legs the visualisation of the tibial-peroneal trunk, anterior tibial artery, posterior tibial artery and fibular artery was recorded in Station IV of the whole body MRA. CTA of the lower leg was not performed.

The data sets were read interactively in 3D mode using both thin slice multi planar reconstructions (MPR) and thin-slab maximum intensity projections (MIP; slab thickness 10 mm). Individual adjustments of the slab thickness were allowed to optimize the visualization of small caliber vessels.

### Statistics

Our null-hypothesis was equivalence of both imaging modalities. To detect differences between MRA and CTA a McNemar test was used for comparison of paired samples. *P* values equal to or smaller than 0.05 were considered significant. All calculations were done using SPSS for Windows 20.0 (IBM, Armonk, NY, U.S.A.).

## Results

All examinations could be performed successfully. No adverse reactions or complications occurred during or after both of the procedures. For all patients CT and MR angiograms could be acquired with a minimum of motion artefact and were of diagnostic quality; therefore no examinations had to be repeated or excluded from the study.

**Figure 1:**
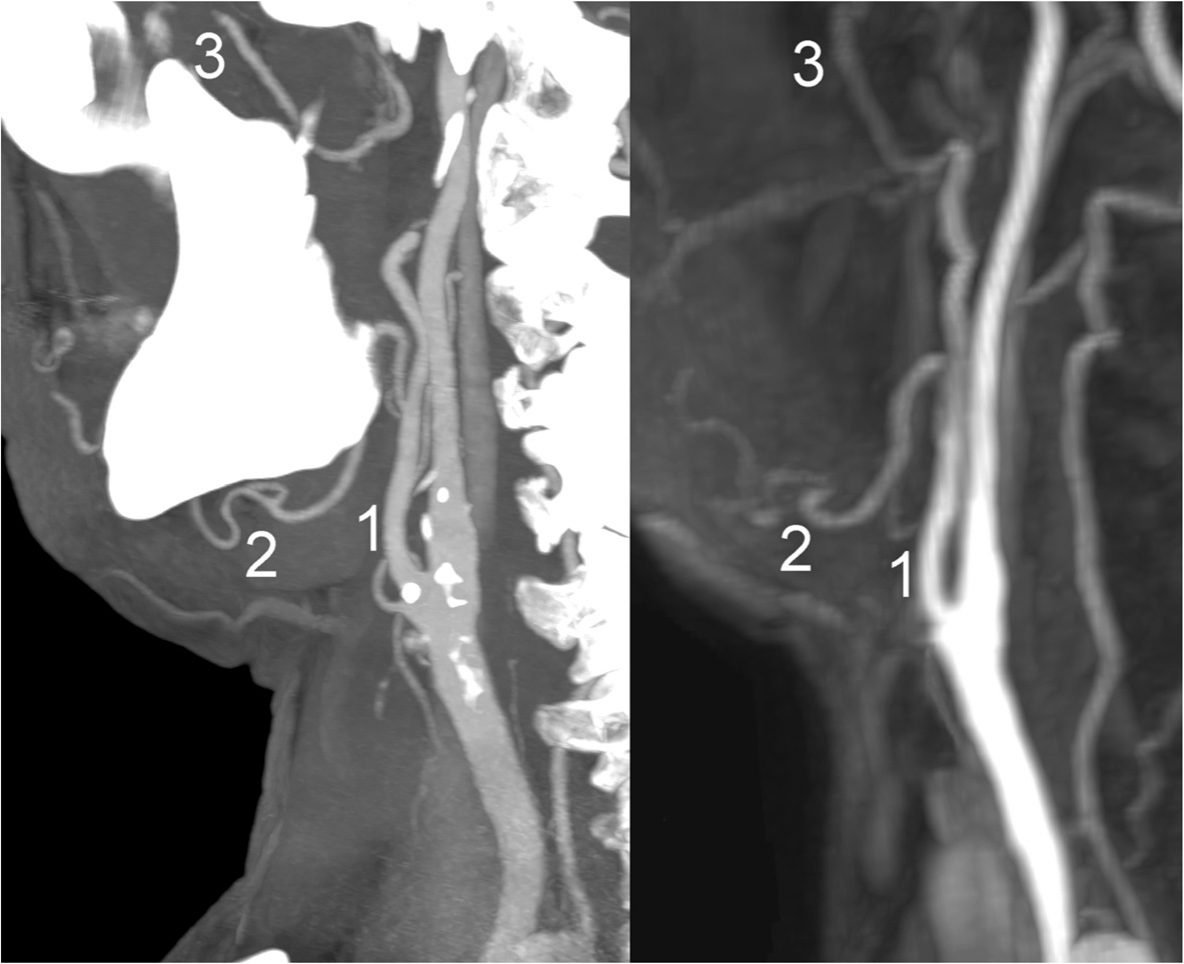
**Sagittal maximum intensity projection (MIP) reconstructions of computed tomography angiography (left) and subtracted magnetic resonance angiography (right) images of the cervical vasculature of a patient scheduled for microvascular reconstruction.** 1: external carotid artery; 2: facial artery; 3: maxillary artery.

**Figure 2:**
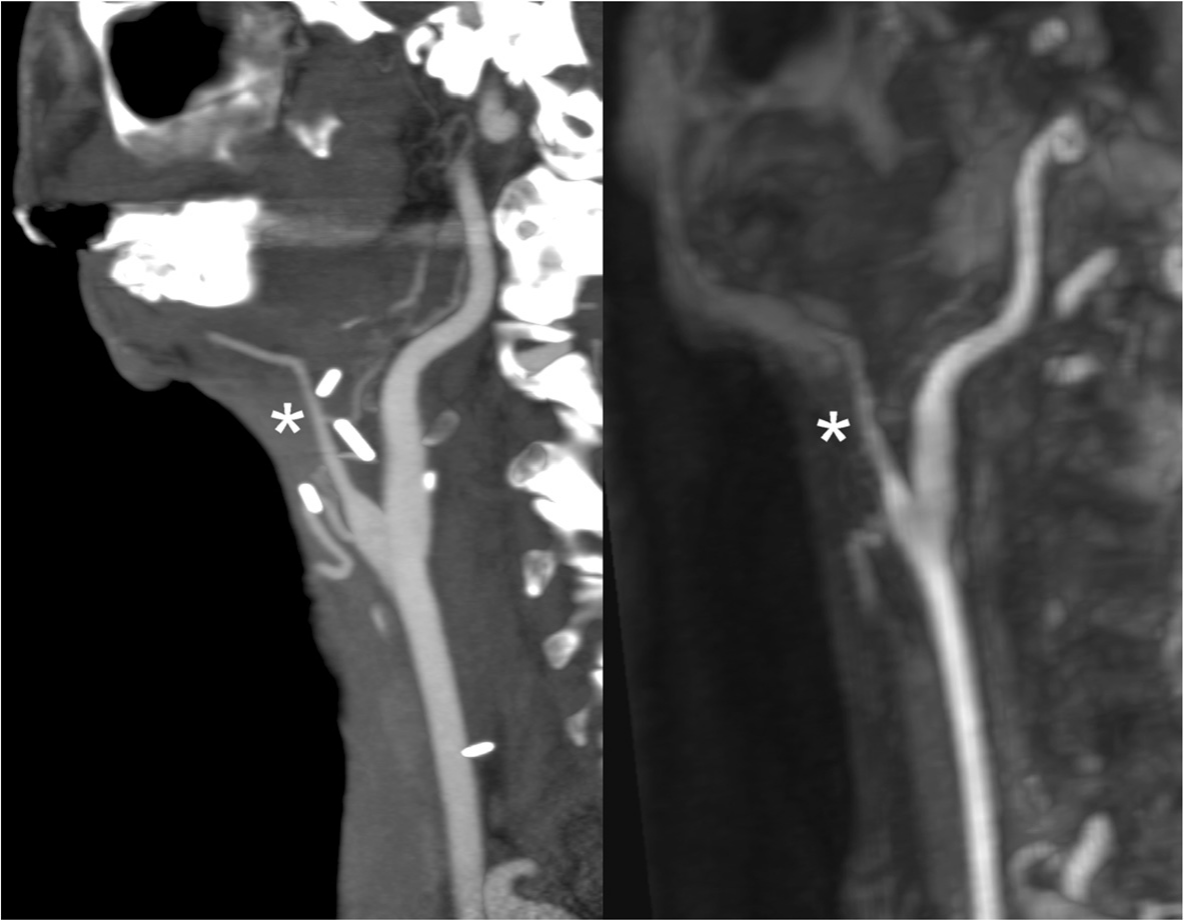
**Sagittal maximum intensity projection (MIP) reconstructions of computed tomography angiography (left) and subtracted magnetic resonance angiography (right) images of a patient who suffers from tumor recurrence and who had previously received a microvascular graft with vessel anastomosis.** The external carotid artery has been resected. Asterisk: graft vessel.

The carotid artery bifurcation was displayed in patients not subjected to prior surgery (Figure 1), as well as in postsurgery (Figure 2) patients. In one patient, with recurrent tumor disease, the mainstem of the external carotid artery was occluded on both sides of the neck, in four patients this vessel was not visualized on one side because of previous surgery. The numbers of branches identified with MRA and CTA respectively are given in Table 2.

**Table 2:** Numbers of arterial branches detected with multi slice computed tomography angiography (CTA) and contrast enhanced magnetic resonance angiography (MRA), in 20 patients (due to previous therapy or atherosclerotic disease the total number of detected vessel segments can be less than 40)

**External carotid artery branches**	**CTA**		**MRA**		***p***
	**Total**	**%**	**Total**	**%**	
Carotid artery bifurcation	40	100	40	100	1.000
External carotid artery mainstem	34	85	34	85	1.000
Superior thyroid artery	37	92.5	37	92.5	1.000
Lingual artery	37	92.5	35	87.5	.500
Facial artery	28	70	28	70	1.000
Ascending pharyngeal artery	33	82.5	3	7.5	< .001
Occipital artery	39	97.5	37	92.5	.500
Posterior auricular artery	33	82.5	16	40	< .001
Superficial temporal artery	36	90	34	85	.500
Maxillary artery	38	95	37	92.5	1.000
All segments	365	91.3	311	77.8	< .001

The tibial-peroneal trunks and fibular arteries in both lower legs were patent in all cases. There was occlusion of the anterior tibial artery in 2 of 40 and occlusion of the posterior tibial artery in 7 of 40 cases.

More vessels were detected with CTA as compared to MRA (*p* < .001). This was especially the case for the smallest vessels, the ascending pharyngeal artery and the posterior auricular artery. When the ascending pharyngeal artery and the posterior auricular artery, vessels not suited for microanastomosis of osteomyocutaneous flaps to reconstruct the mandible, were excluded from the analysis, the difference was no longer statistically significant (p = .118). There was also no statistically significant difference in the subgroup of previously untreated (p = .625) and treated (p = .227) patients.

## Discussion

Prior to head and neck reconstructive surgery using microvascular grafts imaging is warranted to confine the tumor extent, to rule out tumor recurrence, and to assess the vasculature in the host and donor site.

Nonselective digital subtraction angiography (DSA) of the supraaortic vessels is insufficient to display exactly the branches of the external carotid artery; therefore, selective catheter angiography is the modality of choice [6], providing an exquisite spatial resolution and the ability to obtain dynamic information. On the other hand it is an invasive diagnostic procedure that is associated with additional costs as well as significant risks of periprocedural complications [7,8]. Major catheter-associated complications are laceration or dissection of vessels and cerebral embolization by detached plaques or thrombotic material leading to neurological complications (1.3–4.5%) including permanent neurological deficits or death in 0.5–1.3% [9-11].

CTA has been established as a noninvasive standard procedure for a comprehensive imaging of the anatomy of the cervical vasculature. Previous studies comparing DSA with CTA have proved that the latter is a reliable modality in vascular mapping of the cervical vasculature for planning of microvascular reconstructions [2,4,12]. Although modern CT systems allow arterial phase imaging of the neck and lower extremity with only one contrast bolus injection and additional late phase imaging for soft tissue evaluation, CT is at most institutions confined to one body region due to radiation concerns.

Colour-coded duplex ultrasound would be a relatively inexpensive alternative without radiation burden. For carotid artery stenosis high sensitivity and specificity values have been reported [13]. Although the carotid bifurcation can be well visualized, ultrasound does not allow visualization of the complete branching of the external carotid artery, especially in the postoperative, irradiated neck, it is highly operator dependent and allows only segmental views of vascular anatomy rather than longitudinal images.

**Figure 3:**
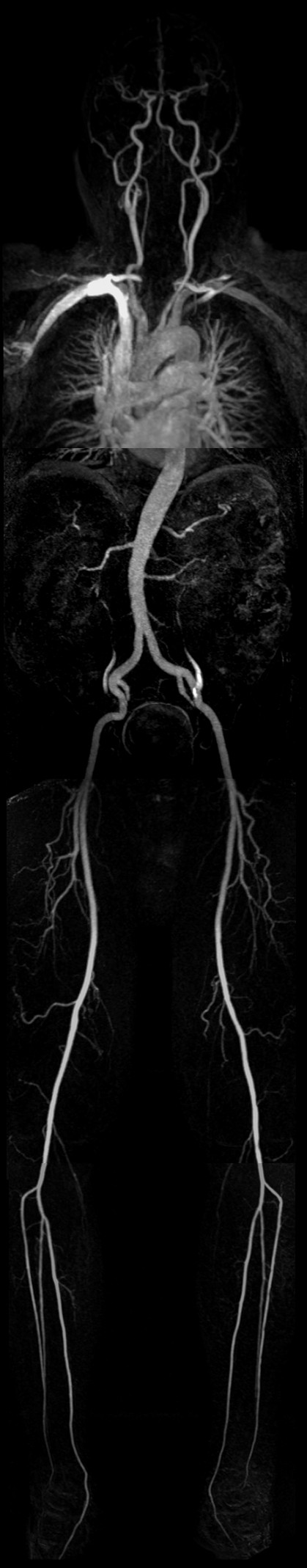
Coronal (MIP) of a subtracted whole-body MRA (two step contrast injection protocol, four overlapping field of views (FOVs)).

Recent developments in MR hard- and software overcame long-standing limitations like reduced longitudinal coverage, insufficient spatial resolution, and long acquisition times. High resolution whole-body MRA became clinically available with modern MR systems and replaced catheter angiography in a variety of indications [14-17]. It has been introduced [5] as a promising tool for preoperative assessment of the arterial system, covering both the donor and host site (Figure 3).

The most feared donor-site complication in fibula flap harvest is foot ischemia secondary to sacrifice of the peroneal artery. In patients with peripheral arterial disease or congenital variants (absent or hypoplastic tibial artery, peroneal artery magna, etc.), the peroneal artery may be the dominant blood supply of the foot [18]. Although only a minority of patients present with congenital variants, atherosclerotic disease is frequently encountered in this population.

The vessels in the host site also need to meet certain preconditions such as equivalent caliber, absence of atherosclerotic or radiation induced stenosis. Excellent results in the detection and grading of carotid artery stenosis have been reported [19-21]. Lohan et al. proposed high filed (3 T) MRA for the evaluation of the external carotid artery and its branches for road mapping surgical and interventional procedures [22]. We sought to evaluate the ability to visualize the donor and host site arterial system with whole-body MRI at 1.5 T. We have chosen a whole-body approach in order to allow identification of alternative donor sites in case fibular harvesting is contraindicated. The MRA protocol we used for the lower extremity has been validated against conventional catheter angiography [23]. The acquisition protocol for the head and neck region had to be adapted in order to include the branches of the external carotid artery. Contrary to Lohan et al. who performed their measurements in a sagittal plane on a 3 T system using an acceleration factor of 6 (phase-encoding direction × 3, slice-encoding direction × 2), we measured the 3D-FLASH sequence in coronal orientation. With this approach we had to focus on the proximal parts of the ECA branches, which are relevant for anastomosis in order to keep imaging time within reasonable limits.

Before this modality could be adopted alone in the clinical routine it had to be tested versus well-established techniques. Unfortunately there exists just limited data on comparison CTA and MRA of the neck [24] and no studies existed that compared the branching of the external carotid artery visualized with whole body MRA to another imaging modality so far. Our study revealed that CTA was superior to MRA when all branches of the external carotid artery were assessed (p < .001). The ascending pharyngeal artery was frequently missed with MRA, presumably due to inferior spatial resolution compared to CTA. When the ascending pharyngeal and posterior auricular artery, arteries which are not relevant for microanastomosis of osteomyocutaneous flaps because of their small caliber and anatomic position, were excluded from the analysis, the differences between CTA and MRA were no longer statistically significant (p = .118). Moreover with the exclusion of these vessels MRA of the head and neck region, being part of a whole-body MRA protocol has demonstrated to be a reliable screening modality for both patients without previous surgery (p = .625) and who were operated or irradiated before (p = .227). This indicates that cervical contrast enhanced MRA can be used as an alternative to CTA, even in the setting of a whole-body angiography prior to free-flap surgery of head and neck. Moreover additional techniques as quantitative MRA could support further advantage of MRA (e.g. to predict the outcome of the implanted grafts) which has to be proven in future studies.

A limitation of our study is that MRA has not been compared to DSA, the standard of reverence. It has been demonstrated, that CTA, the reference we used in our study, is a reliable noninvasive alternative to DSA in the vascular mapping of head and neck [2,4,12], therefore we decided not to expose the patients to the periprocedural risks of DSA. An advantage of CT and MR imaging over DSA is the ability to depict not only the vascular system but also the soft tissues, allowing accurate tumor staging within one imaging session with either modality. However in patients with carcinomas of the oral cavity and oropharynx, metal implants from dental restorations frequently affect image quality of CT studies more than MR studies. As CTA or DSA of the lower legs was not part of the study, no direct comparison of the results of MRA in station IV (lower legs) could be made. However, as MRA of the lower extremity represents a well established diagnostic method, we consider the non-visualisation of the reported vessels in the lower leg as true occlusions. Another limitation lies within the rather low patient number (n = 20). Therefore the statistical results of this study should be confirmed in a follow up study with a larger patient population.

## Conclusions

Our results indicate that whole-body MRA is a promising screening modality for planning of microvascular reconstructions, allowing the identification of relevant host site target vessels in the neck. Therefore it could potentially serve as a radiation free one-stop-shop tool for preoperative assessment of the arterial system, covering both the donor and host site in one single examination.

## Competing interest

The authors declare that they have no competing interests.

## Authors’ contributions

All authors contributed to the conception, design and coordination of the study. MK, SAS, EN, JB, MU, ML made substantial contributions to the acquisition of data and the preparation of the manuscript. MK and SAS drafted and wrote the manuscript. EN, AE, FK, MM, JB, MU, ML revised the manuscript. All authors have given final approval of the version to be published and agree to be accountable for all aspects of the work in ensuring that questions related to the accuracy or integrity of any part of the work are appropriately investigated and resolved.
